# Low expression of SerpinB2 is associated with reduced survival in lung adenocarcinomas

**DOI:** 10.18632/oncotarget.21456

**Published:** 2017-10-03

**Authors:** Maria Ramnefjell, Christina Aamelfot, Lars Helgeland, Lars A. Akslen

**Affiliations:** ^1^ Centre for Cancer Biomarkers CCBIO, Department of Clinical Medicine, Section for Pathology, Haukeland University Hospital, University of Bergen, Bergen, Norway; ^2^ Department of Thoracic Medicine, Haukeland University Hospital, Bergen, Norway; ^3^ Department of Pathology, Haukeland University Hospital, Bergen, Norway

**Keywords:** lung cancer, metastases, SerpinB2, neuroserpin, L1CAM, Pathology Section

## Abstract

Lung cancer is a leading cause of cancer deaths worldwide and new biomarkers are of utmost importance. Studies have indicated that the anti-plasminogen activators SerpinB2 and Neuroserpin, and the adhesion molecule L1CAM, have a coordinated impact on development of metastasis. Here, we examined whether expression of these markers was associated with clinico-pathologic characteristics and prognosis in resected non-small cell lung cancer (NSCLC).

Surgical specimens from 438 NSCLC patients treated at Haukeland University Hospital, Bergen, Norway (1993-2010) were included (median age 68 years; 213 adenocarcinomas, 135 squamous cell carcinomas, 90 others). Representative tumor sections were stained for SerpinB2, Neuroserpin, and L1CAM.

Low expression of SerpinB2 was associated with reduced lung cancer specific survival (LCSS) in adenocarcinomas (*p* = 0.017), also in stage I (*p* = 0.031). In contrast, high SerpinB2 was associated with reduced LCSS in stage I squamous cell carcinomas (*p* = 0.022). Although Neuroserpin and L1CAM showed some associations with clinico-pathologic phenotype, there were no associations with survival. In multivariate survival analysis of adenocarcinomas, low SerpinB2 demonstrated independent prognostic value (HR 1.8, *p* = 0.008).

In summary, low expression of SerpinB2 in lung adenocarcinomas was an independent prognostic factor. In contrast to findings by others, we found no impact of L1CAM on survival. Introduction

## INTRODUCTION

Lung cancer is a leading cause of cancer deaths worldwide [1], and non-small cell lung carcinoma (NSCLC) comprises around 85% of all cases. Dissemination of cancer cells is a critical feature of tumor progression, and the mechanisms responsible for cancer cells being able to invade adjacent structures and form new tumors in other organs are still not fully understood [2]. Finding new biomarkers for predicting disease progress are thus of utmost importance.

In particular, lung and breast cancers account for around two thirds of all metastases to the brain, and these lesions are associated with a high mortality rate [3]. Cells in the brain tissue exhibit several defense-mechanisms to prevent metastasis from forming, and only a limited number of cancer cells survive in this environment [4]. Earlier studies have shown that surviving tumor cells keep in close contact with existing brain capillaries growing as sheaths around them, so-called vascular co-option [5]. To better understand the molecular mechanisms involved, Valiente et al. reported that plasminogen activator inhibitors (SerpinB2 and Neuroserpin) protect cancer cells from an apoptotic cascade initiated by the formation of plasmin in the brain stroma, and along with L1CAM, they are crucial for protecting cancer cells and promoting vascular co-option [6].

SerpinB2 belongs to a superfamily of serpins which act as protease inhibitors (Serine Protease Inhibitors) and has diverse roles in cancer development and metastasis [7]. Serpins inhibit the urokinase plasminogen activator (uPA) and to a certain extent tissue plasminogen activator (tPA). uPA and tPA converts plasminogen to plasmin which in turn aids in the breakdown and remodeling of the extracellular matrix in part through activation of matrix metalloproteinases, thus facilitating invasion and metastasis [8]. The role and expression of plasminogen activator inhibitors 1 and 2 (PAI-1 and PAI-2) have previously been studied in several cancers including lung tumors [9-11], mostly in small cohorts. PAI-1 has not been found to be a prognostic factor for survival in non-small cell lung cancer [12, 13]. SerpinB2, also called PAI-2, is upregulated in activated macrophages, monocytes, fibroblasts, endothelial cells, and differentiating keratinocytes [8]. In smaller studies, low levels of SerpinB2 has been associated with lymph node metastasis and reduced overall survival [11, 14]. However, SerpinB2 expression has not previously been studied in larger cohorts of specific histologic subtypes of NSCLC with long-term follow-up.

Neuroserpin also belongs to the serpin family of serine protease inhibitors, and is expressed mainly in the central nervous system, where it inhibits tPA, thus protecting neurons from the cytotoxic effect of plasmin [15, 16]. Apart from the study by Valiente et al., expression levels of Neuroserpin has not been studied in human cancer [17], and the exact role of Neuroserpin in cancer and metastasis remains to be elucidated.

L1CAM is an adhesion molecule found to be expressed in both neural tissues and tumors in various organs [18-21]. It binds to itself and other proteins through integrins. Recent studies have indicated that L1CAM expression in tumors is associated with poor prognosis [22-24], also in lung cancer [25], and this protein seems to promote tumorigenicity and metastatic potential in NSCLC [26].

Valiente et al. presented these three markers as essential for the formation of brain metastasis from NSCLC, resulting in poor prognosis. Here, we evaluated the expression of SerpinB2, Neuroserpin and L1CAM in a large and population-based cohort of surgically resected primary NSCLC, in order to examine their potential prognostic value, and possible associations with clinico-pathologic characteristics including sites of metastasis. We also aimed to examine the expression of all three markers in matched metastatic lesions, which to the best of our knowledge has not been previously presented.

## RESULTS

### Frequencies

The most common histological diagnosis was adenocarcinoma (49 %), followed by squamous cell carcinoma (31 %) and large cell carcinomas (16 %).

For SerpinB2, 53 % of all cases had high expression, and there was no significant expression difference across the three major histological subgroups (Table 1). Neuroserpin was absent in 25 % of all cases combined, most pronounced among adenocarcinomas (*p* < 0.001) (Table 1). The expression of L1CAM was present in 53 % of all cases, being more frequent in SCC (59 %) and other carcinomas (62 %), compared with 44 % among adenocarcinomas (*p* = 0.003).

**Table 1:** Frequency distribution for SerpinB2, Neuroserpin and L1CAM in 438 cases of non-small cell lung carcinoma

	All cases n=438		AC n=213		SCC n=135		Other NSCLC n=90		p^a^
	n	(%)	n	(%)	n	(%)	n	(%)	
**SerpinB2**									0.205
Low	205^b^	(46.9)	92	(43.2)	71^b^	(53.0)	42	(46.7)	
High	232	(53.1)	121	(56.8)	63^b^	(47.0)	48	(53.3)	
**Neuroserpin**									<0.001
Absent	108	(24.7)	73	(34.3)	16	(11.9)	19	(21.1)	
Present	330	(75.3)	140	(65.7)	119	(88.1)	71	(78.9)	
**L1CAM**									0.003
Absent	208	(47.5)	119	(55.9)	55	(40.7)	34	(37.8)	
Present	230	(52.5)	94	(44.1)	80	(59.3)	56	(62.2)	

^a^p-values from Pearson’s chi-square test across the three subgroups; ^b^1 case missing (staining)

n, number of patients; AC, adenocarcinoma; SCC, squamous cell carcinoma; NSCLC, non-small cell lung carcinoma

### Associations in primary tumors

Expression of SerpinB2 was not associated with any of the basic clinico-pathologic features when all cases were combined, or in subgroup analysis (Supplementary Table 1). For all cases combined, Neuroserpin associated with larger tumor diameter, presence of necrosis, and pleural invasion (p<0.05 for all) (Supplementary Table 2). In the subgroup of AC, there were positive associations with BVI, necrosis, and pleural invasion (p<0.05 for all). For SCC and other carcinomas, no associations with basic variables were found. For all cases, L1CAM expression was associated with high histologic grade, BVI, LVI, and necrosis (p<0.05 for all) (Supplementary Table 3). For the subgroup of AC, the same pattern was seen with associations to high histologic grade, BVI, and necrosis, as well as with pleural invasion, and tumor stage (p<0.05 for all). In SCC, presence of L1CAM was associated with high histologic grade (*p* = 0.039). In other NSCLC, no significant associations with basic variables were found.

### Metastasis

For SerpinB2, we found no apparent associations with clinically or histologically documented presence of metastasis to any sites during the follow-up period (Supplementary Table 4), and few associations were present for Neuroserpin (Supplementary Table 5). L1CAM was not related to presence of metastasis at any site during follow-up when all cases were included, although there was a significant association in the subset of AC with occurrence of liver metastasis, OR 3.99 (95% CI 1.38 - 11.52; *p* = 0.007) (Supplementary Table 6).

When we stained tissues from metastatic lesions from 43 paired cases (Supplementary Table 7), we found no correlations between the staining index in primary tumors (dichotomized as described in Evaluation of staining) and matched metastatic lesions (by McNemars test), for any of the three markers. Further, there were no significant differences in the level of staining (by the staining index) for the three markers between all primary tumors and all metastases (by Wilcoxon Signed Rank test).

When we compared the staining index (dichotomized by SI = 4) between different metastatic sites, low SerpinB2 expression was present in 64 % (7/11) of the brain metastases, compared with 28 % (9/23) of the other metastases combined (*p* = 0.068, Pearson’s chi-square test) (Supplementary Table 8A and 8B).

### Univariate survival analysis

#### Adenocarcinoma

**Figure 1:**
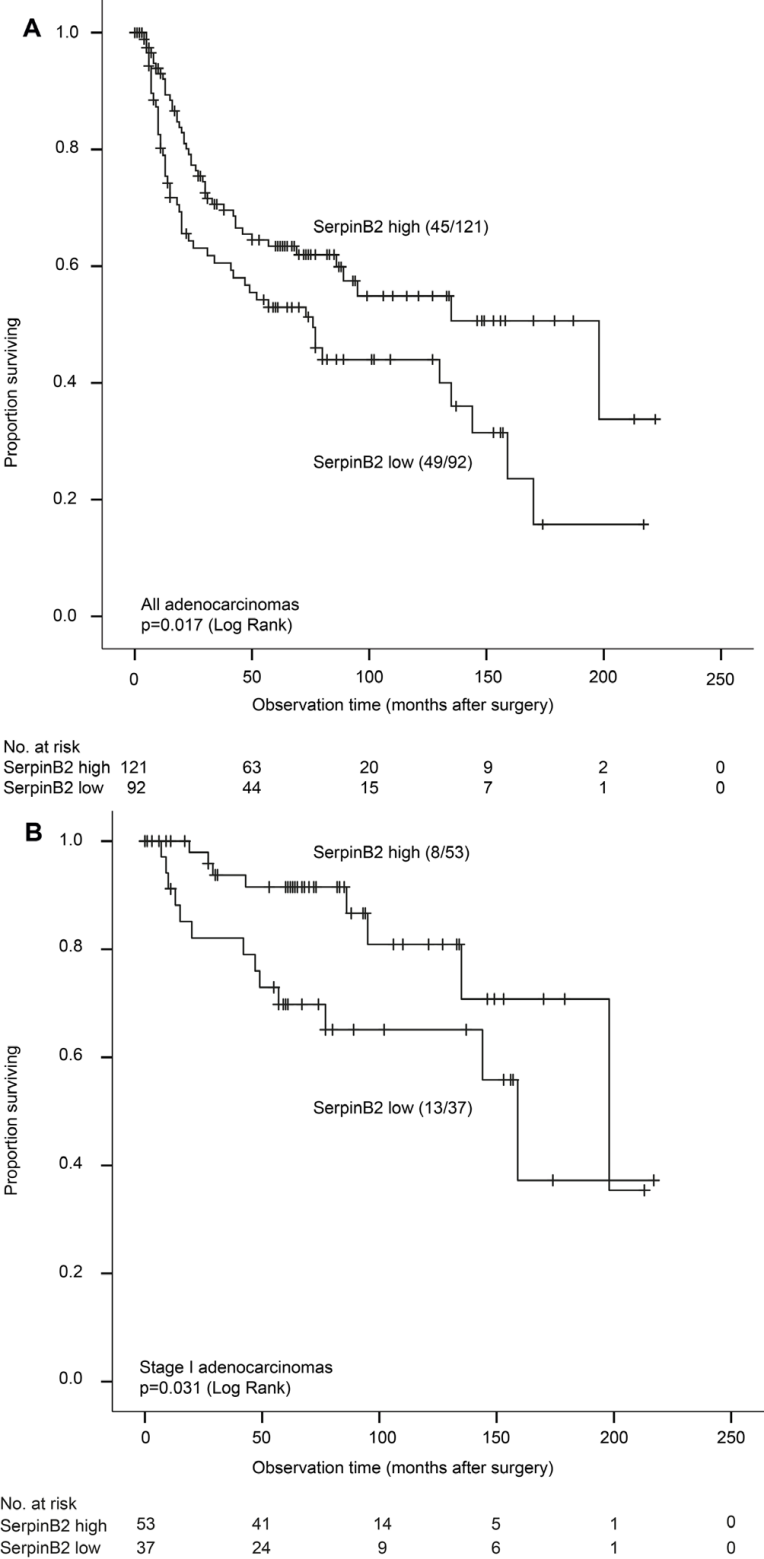
**A.**Lung cancer specific survival for adenocarcinomas (*n* = 213) according to low and high expression of SerpinB2 (Kaplan-Meier). Numbers in brackets indicate events and total number of cases in each group. **B.** Lung cancer specific survival for stage I adenocarcinomas (*n* = 90) according to low and high expression of SerpinB2 (Kaplan-Meier). Numbers in brackets indicate events and total number of cases in each group.

Low expression of SerpinB2 in primary tumors was associated with reduced LCSS, p = 0.017 (Figure 1A). Median survival time was 76 months in cases with low SerpinB2 expression (by median value) and 198 months for cases with high expression. A significant difference was also found for disease-free survival (DFS) (*p* = 0.023; median survival 41 versus 63 months, respectively). Among stage I adenocarcinomas, low SerpinB2 expression was still associated with reduced LCSS (p = 0.031) (Figure 1B).

No significant differences in LCSS or disease-free survival were found for Neuroserpin or L1CAM for all adenocarcinoma cases (Supplementary Table 9). For Neuroserpin, this did not change when we used median staining index as cut-off value (SI = 2). Subclassification of adenocarcinomas in subtypes (by IASLC or WHO) was not a significant prognostic factor (data not shown). Receiving adjuvant chemotherapy or radiotherapy was associated with reduced LCSS (*p* = 0.025 and *p* < 0.001, respectively). Also, smoking history (never versus ever) was not significant in univariate analysis (HR 0.7; 95% CI 0.38-1.34, *p* = 0.29).

#### Squamous cell carcinoma and other NSCLC

There were no differences in LCSS or DFS for SerpinB2, Neuroserpin and L1CAM expression in SCC and other NSCLC (Supplementary Table 9). For Neuroserpin, this did not change when we used median staining index as cut-off value (SI = 2). When analyzing stage I SCC separately, high SerpinB2 expression (by median value) was associated with reduced LCSS (*p* = 0.022) (Supplementary Figure 1) and DFS (*p* = 0.044).

### Multivariate survival analysis

In a multivariate model for AC where sex, histologic grade (high versus low), blood vessel invasion (present versus absent), lymphatic vessel involvement (present versus absent), necrosis (present versus absent), tumor stage (II-IV versus I), and SerpinB2 expression (low versus high) were included, low SerpinB2 demonstrated independent prognostic value for LCSS (HR 1.8 (95% CI 1.17-2.06), *p* = 0.008) (Table 2). In the final model for stage I adenocarcinomas, SerpinB2 retained independent prognostic value for LCSS (HR 2.6 (95 % CI 1.03-6.58), *p* = 0.043) (Supplementary Table 10A).

**Table 2:** Univariate and multivariate survival analysis (Cox´ proportional hazards method) of adenocarcinomas (n=213) with regards to lung cancer specific survival

Variables	Categories	Univariate			Multivariate		
		HR	(95% CI)	p	HR	(95% CI)	p
**Age**	≤68 years	1.0			NI		
	>69 years	1.2	(0.79-1.77)	ns			
**Sex**	Female	1.0			1.0		
	Male	2.3	(1.53-3.58)	<0.001	2.7	(1.73-4.26)	<0.001
**SerpinB2**	High	1.0			1.0		
	Low	1.6	(1.09-2.44)	0.018	1.8	(1.17-2.06)	0.008
**Histologic grade**	Low	1.0			1.0		
	High	1.6	(1.09-2.46)	0.017	1.3	(0.87-2.09)	ns
**Blood vessel invasion**	Absent	1.0			1.0		
	Present	2.2	(1.42-3.27)	<0.001	1.5	(0.98-2.43)	0.060
**Lymph vessel invasion**	Absent	1.0			1.0		
	Present	2.7	(1.79-4.15)	<0.001	1.6	(1.02-2.58)	0.042
**Necrosis**	Absent	1.0			1.0		
	Present	2.1	(1.39-3.32)	0.001	1.4	(0.90-2.30)	ns
**Tumor stage**	I	1.0			1.0		
	II-IV	4.3	(2.63-7.18)	<0.001	3.2	(1.83-5.53)	<0.001

HR, hazard ratio; CI, confidence interval; NI, not included; ns, not significant (p<0.10)

In stage I squamous cell carcinomas, a multivariate model where variables with p<0.10 in univariate survival analysis (BVI and SerpinB2) were included, showed that high SerpinB2 was an independent prognostic factor for reduced LCSS (HR 3.5 (95 % CI 1.03-11.74), *p* = 0.044) (Supplementary Table 10B).

In survival analyses, exclusion of patients who died within 1 month after surgery (*n* = 7) did not change the results of multivariate survival analyses (data not shown), nor did the inclusion of adjuvant therapy (data not shown).

## DISCUSSION

Recently, SerpinB2, Neuroserpin and L1CAM were reported to be key drivers in experimental studies of lung cancer dissemination, with special focus on the biology of brain metastases [6]. The authors used mRNA levels of SerpinB2 and Neuroserpin in survival analyses of 106 primary lung adenocarcinomas with brain metastasis as end-point. Further, protein status by immunohistochemistry showed prevalent expression of these markers in a series of 33 human brain metastatic lesions. Here, in this first study performed on human NSCLC, and using a large cohort with long follow-up, we aimed to examine whether the expression of these three protein markers in the primary lung tumors were associated with prognostic value, using cancer specific survival as the end-point.

Our main finding is that low SerpinB2 expression in primary tumors is independently associated with reduced cancer specific survival in lung adenocarcinomas. This study of SerpinB2 is the first to include multivariate survival analysis of specific histologic subtypes of NSCLC, and the largest cohort to our knowledge. Our findings support data from model studies that SerpinB2 (PAI-2) is an important regulator of lung cancer progression [6, 27]. Further, this is also the first paper to address the protein expression of SerpinB2, Neuroserpin and L1CAM in primary resected NSCLC and their matched metastatic lesions.

Notably, our finding that low expression of SerpinB2 is associated with reduced cancer specific survival is in line with previous lung cancer studies where low tumor cell expression of SerpinB2 was associated with nodal metastasis [11] and tumor dissemination [14].

The biological function of SerpinB2 is known to some extent, but its complete role in human cancer progression, and in different tumor types, is controversial [28, 29]. It has been proposed as an inhibitor of plasmin-formation with effects on matrix metalloproteinases, possibly inhibiting angiogenesis and invasion that could lead to reduced survival [30, 31]. Thus, low SerpinB2 could potentially be associated with increased tumor progress by angiogenesis and tumor invasion. Regarding tumor associated angiogenesis, it should be mentioned that the influence of anti-angiogenic treatment to reduce growth and spread of malignant tumors has been challenged, with the possibility of paradoxic and stimulatory influences instead [32, 33]. In our study, the direct relationship between the three markers and angiogenesis indicators was not examined.

Whereas low SerpinB2 expression was prognostic for survival in adenocarcinomas, there were no survival differences in SCC or other NSCLC when all stages were included. Still, when stage I tumors were analyzed separately, opposing results for AC and SCC appeared. This difference in prognostic impact has not been presented before, and potentially reflects differences in how SerpinB2 affects tumor biology in AC and SCC at the early stages. Different biological properties could also explain some of the controversial results of SerpinB2 as a prognostic marker in other solid cancers. Whereas high SerpinB2 was related to reduced survival in bladder cancer [34], endometrial cancer [35], and esophageal squamous cell carcinoma [36], low SerpinB2 has been associated with worse survival in breast cancer [37, 38], hepatocellular carcinoma [39], and pancreatic cancer [40].

Lung adenocarcinoma and squamous cell carcinomas have been extensively characterized at the molecular level [41, 42], and these types show different genomic profiles [43]. Additionally, lung squamous cell carcinoma has been shown to share molecular traits with bladder carcinomas [44]. Genomic characteristics in lung carcinoma subgroups could possibly explain differences in the expression and prognostic effect of proteolytic enzymes and their inhibitors. SerpinB2 acts via a covalent binding to uPAR and thereby inhibits plasmin formation through inhibition of plasminogen activator [8]. This is a complicated system that also involves matrix metalloproteinases (MMPs). One study comparing different subgroups where high MMP-9 expression in tumor cells indicated worse outcome in resected lung adenocarcinomas, but not in lung squamous cell carcinomas, has been reported [45], thus further pointing to differences in biology between the subgroups. The exact mechanisms of SerpinB2 function in different cancer types is to date not fully understood [7].

Whereas Valiente et al. reported prevalent expression of SerpinB2, Neuroserpin and L1CAM in brain metastatic lesions (33 lesions for SerpinB2 and Neuroserpin; 31 lesions for L1CAM) [6], we could not directly reproduce these results. On the contrary, we found a trend towards lower expression of SerpinB2 in human brain metastases, compared to other metastatic sites combined. The reason for this is not clear. Regarding staining evaluation, Valiente et al. dichotomized cases of SerpinB2 and Neuroserpin into positive when more than 80 % of cells were stained without considering staining intensity and negative when less than 80 % of cells had visible staining. We applied a staining index, where both the intensity of staining as well as the area of stained cells were taken into account, using the median (SI=4) as cut-off value between low and high SerpinB2 expression.

The discordant expression of these markers in primary and secondary tumors could in part be explained by tumor heterogeneity which has been focused lately [46-49]. With the advancement of molecular studies and gene sequencing, the issue of tumor heterogeneity has become even more complicated [47, 50].

There are certain limitations to our study since it is based on a retrospective cohort. However, we included all patients eligible for surgery during the time period. Our hospital is the main referral center for thoracic surgery in our county, which comprises around 10 % of the national population. As such, our cohort can be regarded as representative. We included all non-small cell lung cancers, and to avoid too small subgroups in statistical analysis, we decided to combine large cell carcinoma, large cell neuroendocrine carcinoma and sarcomatoid carcinomas in the group ‘other NSCLCs’. This combination may contribute to the lack of significant results in this heterogeneous group, although a more detailed study of the small subgroups did not reveal major differences from the group as a whole (data not shown). Another limitation is the lack of an independent but similar validation cohort, and this is warranted in further studies of such biomarkers. As an additional limitation, we did not add special stains for the evaluation of basic tumor characteristics such as pleural invasion.

Regarding the occurrence of metastases at different sites, clinical records might not be complete with respect to such information, and this could be a potential bias in our analyses. We acknowledge that the lack of significant associations to metastasis can be considered a limitation to our results on survival. However, we believe that disease specific survival is a robust and informative end-point. Finally, time to metastatic event at different sites was not available, and this could possibly reduce the sensitivity of the data we have presented.

In summary, as a novel finding, low SerpinB2 expression in lung adenocarcinomas was independently associated with reduced lung cancer specific survival in our cohort. This was also found in stage I adenocarcinomas, and SerpinB2 levels could potentially help stratify patients for closer follow-up and adjuvant therapy. In contrast to findings by others, we did not observe a prognostic impact of L1CAM. Thus, low expression of SerpinB2 could be a biomarker for prognosis in lung adenocarcinomas.

## MATERIALS AND METHODS

### Patients

Formalin-fixed and paraffin-embedded (FFPE) tissue of surgically resected NSCLC from 450 patients diagnosed during 1993-2010 was retrieved from the archives at the Department of Pathology, Haukeland University Hospital, Bergen, Norway. All patients surgically treated for NSCLC at Haukeland University Hospital (the main referral center in our region) in the time-period were included. All cases were reviewed by two pathologists (M.R. and L.H.). Twelve cases were excluded (revised diagnosis, missing blocks, lack of follow-up information), leaving 438 patients for further studies (median age 68 years; 63% males).

We also retrieved metastatic lesions from the archives, and found matching tissue from a total of 60 cases. In 43 of these there was sufficient FFPE material for further analysis.

### Variables recorded

Histological type and grade were determined according to the World Health Organization (WHO) [51]. All evaluations were done in 2014, and as such the 2004 classification was used. During re-assessment, 21 cases needed additional immunohistochemistry to confirm histologic type. Regarding grade, the well and moderately differentiated tumors were combined into a low-grade group, whereas poorly differentiated and undifferentiated tumors were combined into a high-grade group. We added the proposal from the International Association for the Study of Lung Cancer (IASLC) [52] with regards to the predominant growth pattern for adenocarcinoma (AC). Apart from AC and squamous cell carcinoma (SCC), we included large cell carcinoma, large cell neuroendocrine carcinoma, adenosquamous carcinoma, and sarcomatoid carcinoma in a group of other NSCLC. Stage was determined using the TNM Classification of Malignant Tumors (7^th^ edition) [51].

From the clinical records we recorded the following information: sex, age at surgery, fixation time (in days), type of specimen (segment, lobectomy, others), tumor localization, tumor size (defined as largest diameter measured macroscopically), number of tumor blocks, adjuvant treatment (chemo- or radiotherapy), time of first disease recurrence, type of first recurrence (local, regional, distant), sites of metastasis during follow-up (liver, adrenals, brain, bone, skin and others like intrathoracic sites, distant lymph nodes, and kidneys, by clinical ascertainment), time of death, and cause of death. Further, blood vessel invasion (BVI), lymphatic involvement (LVI), pleural invasion, necrosis and inflammation were recorded from routine Hematoxylin & Eosin stained sections without the use of additional staining as we previously reported [53]. Briefly, inflammation was recorded subjectively by evaluating the infiltration of lymphocytes and plasma cells in and between tumor cell nests. Cases with scattered lymphoid cells were regarded as having mild inflammation. Cases with moderate inflammation had denser and fused infiltrates between tumor cells and scattered lymphoid cells within tumor cell aggregates. Marked inflammation was recorded when there was dense infiltration of lymphocytes and plasma cells both within and around tumor cell nests. Similar approaches have previously been described [54, 55].

### Immunohistochemistry

Selected tumor blocks were stained for SerpinB2, Neuroserpin and L1CAM using standard immunohistochemical protocols. Briefly, 4 μm sections were deparaffinised in xylene and rehydrated in alcohol and distilled water. Target retrieval was obtained by boiling in buffered solution at pH6 (Dako S1699 for Neuroserpin and L1CAM) or at pH9 (Dako 2367 for SerpinB2) using a microwave oven for 20 minutes. After 20 minutes cooling, distilled water was added to reduce the fluid to room temperature. Peroxidase block (Dako 2023) was added for 8 minutes to block endogenous peroxidase, followed by protein-block (Dako X0909) for 8 minutes. Buffered saline solution (Dako S3006) was used in between steps. Incubation with primary antibody was performed at room temperature for 60 minutes (Neuroserpin, Anti-Serpini1 (HPA001565), Sigma Aldrich, dilution 1:100), or overnight at 4°C (SerpinB2, PAI-2 (H70), Santa Cruz, sc-25745 at dilution 1:200 and L1CAM, Sigma Aldrich, Sig-3911, at dilution 1:100). A HRP-labelled polyclonal anti-rabbit polymer (Dako K4011 for Neuroserpin and SerpinB2) and monoclonal anti-mouse (Dako K4007 for L1CAM) secondary antibody was added for 30 minutes at room temperature. DAB (Dako K4007) was used as a chromogen, and haematoxylin (Dako S2020) as a counterstain. For Neuroserpin, an autostainer (Dako Autostainer, serial number 3400-9567) was used. Concerning SerpinB2 and L1CAM, a humidifying chamber (Magnetic Immuno Staining Tray, CellPath, UK) was used. Positive controls (human placenta for SerpinB2, brain parenchyma for Neuroserpin, and stomach wall for L1CAM) were included in every run.

Regarding choice of antibodies, our aim was to validate the results by Valiente et al. [6]. As such, we wanted to use the same antibodies as these authors, and we therefore decided to apply polyclonal antibodies for Neuroserpin and SerpinB2, although we are aware of the limitations of polyclonal as compared to monoclonal antibodies. For Neuroserpin, the antibody used by Valiente et al. was no longer available according to the manufacturer, and we selected an alternative antibody based on recommendations in The Human Protein Atlas (http://www.proteinatlas.org).

### Evaluation of staining

**Figure 2:**
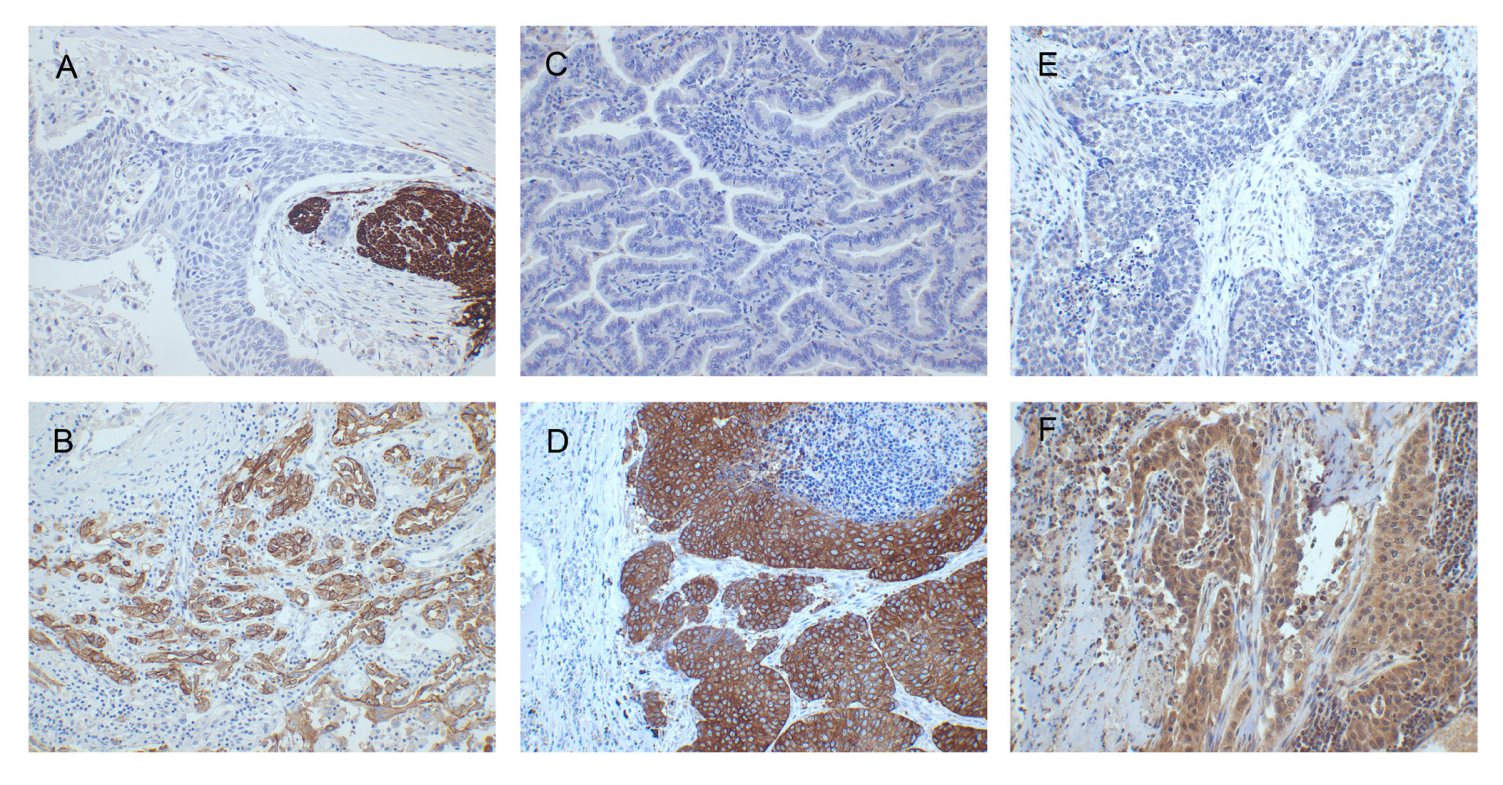
Immunohistochemistry (magnification x200) **A.** L1CAM absent (staining index 0, positive internal control in peripheral nerve); **B.** L1CAM present (staining index 9); **C.** Neuroserpin absent (staining index 0); **D.** Neuroserpin present (staining index 9); **E.** weak SerpinB2 expression (staining index 2); **F.** moderate SerpinB2 expression (staining index 6).

All slides were examined by one senior lung pathologist (M.R.) blinded to patient information, and some difficult cases were discussed. Immunohistochemical staining of the candidate markers was evaluated using a staining index where staining intensity in tumor cells (score 0-3) is multiplied by the area or proportion of stained tumor cells (score 1; <10 %, score 2; 10-50 %, and score 3; >50 %). For SerpinB2 and Neuroserpin, cytoplasmic staining was considered based on previous studies [36, 56], and evaluated as absent, weak, moderate or strong (0-3). For L1CAM staining intensity, based on previous studies with minor modifications [25], cytoplasmic staining was regarded as weak (score 1), partial membranous staining as moderate (score 2) and complete membranous staining as intense (score 3) (Figure 2). The intensity of staining was evaluated and compared to the positive controls for each biomarker.

SerpinB2 showed a homogenous, cytoplasmic staining in tumor cells. In some cases, nuclear staining was also noted. Alveolar macrophages served as an internal positive control when present as these were strongly stained. We also noted weaker staining of stromal fibroblasts, and these were clearly separate from tumor cells. Neuroserpin showed cytoplasmic staining in tumor cells without expression in non-tumor cells. The staining intensity was compared to the positive control in brain parenchyma included in every run. There were also cases with a heterogeneous staining pattern, showing nest of positive tumor cells in some areas. L1CAM showed both cytoplasmic and membranous staining in tumor cells, with positive internal control in peripheral nerves.

There are no validated cut-off values for any of the three candidate markers. By default, median and quartile values were examined, along with frequency distribution for the biomarkers with number of events in potential subgroups, prior to final statistical analysis including survival studies. We aimed to select robust cut-off values (such as the median) to secure sufficient statistical power in subgroup analyses. In contrast, we did not search for optimal cut-off values, to avoid overfitting of the data. Thus, for SerpinB2, the staining index nearly followed a normal distribution, and the median value was selected as cut-off value (SI=4). All cases with SI<4 were grouped as having low expression, and those with SI≥4 as having high expression. For Neuroserpin, the median was at staining index 2. However, here we also included a qualitative evaluation of the staining pattern, which was heterogeneous in some cases with nests of positive tumor cells among negative areas. In order to include cases with this mosaic pattern as positive, we decided to set the cut-off value at staining index 1. For L1CAM, the distribution was skewed to the left with a high number of negative cases (i.e. staining index 0), and the median value was at staining index 1 which was set as the cut-off value.

### Follow-up

Follow-up was completed by May 16^th^ 2015. Median follow-up was 82 months (range 1-254). At latest follow-up, 177 (40 %) had died from lung cancer, 135 (31 %) had died from other causes, and 118 (27 %) patients were recovered. Eight patients (2 %) were alive with disease recurrence (Table 3).

**Table 3:** Demographic and histologic data for major subgroups of non-small cell lung carcinoma (n=438)

	AC (n=213)		SCC (n=135)		Other NSCLC (n=90)		*p*
	n	(%)	n	(%)	n	(%)	
**Sex**							<0.001^a^
Male	111	(52.1)	109	(80.7)	57	(63.3)	
Female	102	(47.9)	26	(19.3)	33	(36.7)	
**Age**							ns
Median (range)	67	(32-84)	69	(24-84)	66	(44-85)	
**Tumor size**							<0.001^b^
Median (range)	30	(10-130)	40	(7-110)	40	(12-165)	
**Histologic grade**							<0.001^a^
Well differentiated	33	(15.5)	6	(4.4)	0	(0)	
Mod. differentiated	82	(38.5)	63	(46.7)	4	(4.4)	
Poorly differentiated	97	(45.5)	66	(48.9)	12	(13.3)	
Undifferentiated	1	(0.5)	0	(0)	74	(82.2)	
**Adenocarcinoma subtype**							
MIA	2	(0.9)					
Lepidic	20	(9.4)					
Acinar	82	(38.5)					
Papillary	20	(9.4)					
Micropapillary	3	(1.4)					
Solid	81	(38.0)					
Mucinous	5	(2.3)					
**BVI**							0.029^a^
Absent	149	(70.0)	111	(82.2)	70	(77.8)	
Present	64	(30.0)	24	(17.8)	20	(22.2)	
**LVI**							0.023^a^
Absent	158	(74.2)	112	(83.0)	78	(86.7)	
Present	55	(25.8)	23	(17.0)	12	(13.3)	
**Necrosis**							<0.001^a^
Absent	94	(44.1)	9	(6.7)	7	(7.8)	
Present	119	(55.9)	125	(92.6)	83	(92.2)	
**Inflammation**							0.003^a^
Mild/moderate	192	(90.1)	104	(77.0)	73	(81.1)	
Severe	21	(9.9)	31	(23.0)	17	(18.9)	
**Pleural invasion**							0.031^a^
No	149	(70.0)	111	(82.2)	64	(71.1)	
Yes	64	(30.0)	24	(17.8)	26	(28.9)	
**Tumor stage**							ns
I	90	(42.3)	62	(45.9)	36	(40.0)	
II	72	(33.8)	51	(37.8)	32	(35.6)	
III	42	(19.7)	20	(14.8)	19	(21.1)	
IV	5	(2.3)	2	(1.5)	0	(0)	
Unknown	4	(1.9)	0	(0)	3	(3.3)	
**Sites of metastasis during follow-up**							
Liver	19	(8.9)	9	(6.7)	13	(14.8)	ns
Adrenal	16	(7.5)	0	(0.0)	7	(5.3)	0.004
Brain	47	(22.1)	8	(6.0)	18	(20.2)	<0.001
Bone	36	(16.9)	13	(9.7)	13	(14.6)	ns
Skin	7	(3.3)	4	(3.0)	2	(2.2)	ns
Other	28	(13.1)	9	(6.7)	10	(11.2)	ns
**Status at latest observation**							0.016^a^
Dead from lung cancer	94	(44.1)	43	(31.9)	40	(44.4)	
Dead with lung cancer	8	(3.8)	3	(2.2)	4	(4.4)	
Dead from other causes	44	(20.6)	53	(39.2)	23	(25.6)	
Alive with disease recurrence	7	(3.3)	0	(0)	1	(1.1)	
Recovered	60	(28.2)	36	(26.7)	22	(24.4)	
**Smoking history**							0.021
Never	20	(9.4)	1	(0.7)	3	(3.3)	
Former	76	(35.7)	56	(41.8)	34	(37.8)	
Current	116	(54.5)	75	(56.0)	51	(56.7)	
Unknown	1	(0.5)	2	(1.5)	2	(1.1)	
**Adjuvant radiotherapy**							ns
No	180	(84.5)	119	(88.1)	77	(85.6)	
Yes	30	(14.1)	14	(10.4)	12	(13.3)	
Unknown	3	(1.4)	2	(1.5)	1	(1.1)	
**Adjuvant chemotherapy**							ns
No	159	(74.6)	103	(76.3)	69	(76.7)	
Yes	51	(23.9)	30	(22.2)	20	(22.2)	
Unknown	3	(1.4)	2	(1.5)	1	(1.1)	

^a^Pearson’s chi-square test across the three histological subgroups^**b**^Independent samples Kruskal-Wallis test across the three histological subgroups

AC, adenocarcinoma; SCC, squamous cell carcinoma; n, number of patients; BVI, blood vessel invasion; LVI, lymphatic vessel invasion; ns, not significant

In the follow-up period, 211 (48 %) patients developed metastatic disease. Median recurrence-free time was 13 months (range 0-198 months). The most common first site of recurrence was metastasis to a distant tissue, *n* = 122 (28 %), whereas fewer patients had their first recurrence at a local or regional site, *n* = 53 (12 %) and *n* = 36 (8 %). The most common metastatic site was the brain (73 patients), followed by bone (62), liver (41), adrenals (23), skin (13) and other sites (intrathoracic, distant lymph nodes, kidneys, spleen, gastrointestinal) (47).

### Statistical methods

For associations between categorical data the Pearson’s chi-square test was performed, and odds ratios (OR) were computed. For continuous variables, the independent samples Mann-Whitney U-test and Kruskal-Wallis test were used. Correlations between ordinal data were tested by the Spearman rank correlation test. For related samples, the McNemar test and Wilcoxon Signed Rank test were applied. End-points were lung cancer specific survival (LCSS), recorded as the time from surgery to the time of death from lung cancer. Patients alive at last follow-up or dead from other causes were censored. Disease-free survival (DFS) was recorded as time from surgery to time of recurrent disease. For survival analysis, Kaplan-Meier curves were computed and differences between groups were analyzed using the log-rank test. The Cox´ proportional hazards method was used for multivariate analysis including variables with p<0.10 in univariate analysis. Assumptions of proportionality were checked by using the Nelson-Aalen method for categorical data, and the scatter plot method for continuous data. Interactions were tested by applying the product-term method. Significance was set at p-values < 0.05. The statistical analyses were performed using SPSS (IBM, version 22-24).

### Ethics

The study was approved by the Regional Ethics Committee (#2013-529 REK Nord). The study was exempt from informed consent.

## SUPPLEMENTARY MATERIALS FIGURE AND TABLES





## Abbreviations

AC: Adenocarcinoma
BVI: Blood vessel invasion
CI: Confidence interval
DFS: Disease free survival
IASLC: International Association for the Study of Lung Cancer
LCSS: Lung cancer specific survival
LVI: Lymph vessel invasion
NSCLC: Non-small cell lung carcinoma
PAI: Plasminogen activator inhibitor
SCC: Squamous cell carcinoma
TNM: Tumor Node Metastasis
tPA: Tissue plasminogen activator
uPA: Urokinase plasminogen activator
WHO: World Health Organization
