# Supplementary material for: Low expression of SerpinB2 is associated with reduced survival in lung adenocarcinomas
Source: Oncotarget. 2017 Oct 3;8(53):90706–18. doi: 10.18632/oncotarget.21456 (PMC5710879; doi:10.18632/oncotarget.21456)
Supplement: Supplementary file 1 [file oncotarget-08-90706-s001.pdf]

Low expression of SerpinB2 is associated with reduced survival in lung adenocarcinomas

SUPPLEMENTARY MATERIALS

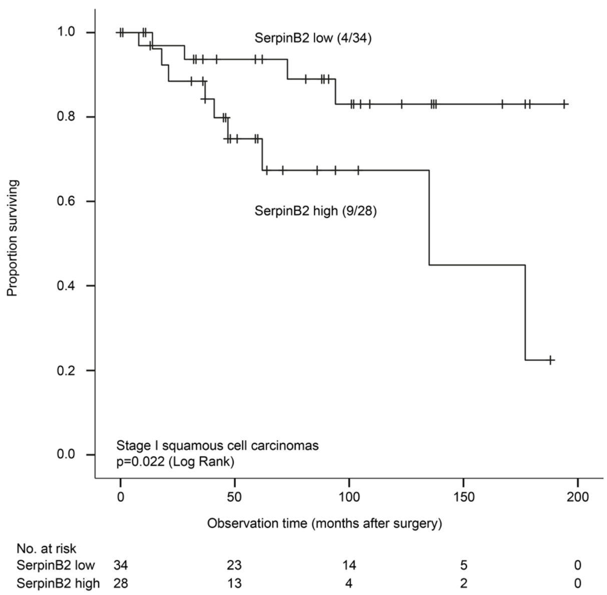

Supplementary Figure 1: Lung cancer specific survival for stage I squamous cell carcinomas (n=62) according to low and high expression of SerpinB2 (Kaplan-Meier). Numbers in brackets indicate events and total number of cases in each group.

For Supplementary Tables, see Supplementary Tables files
