# Supplementary material for: Low expression of SerpinB2 is associated with reduced survival in lung adenocarcinomas
Source: Oncotarget. 2017 Oct 3;8(53):90706–18. doi: 10.18632/oncotarget.21456 (PMC5710879; doi:10.18632/oncotarget.21456)
Supplement: Supplementary file 2 [file oncotarget-08-90706-s002.docx]

**Supplementary Table 1: Associations between SerpinB2 expression (low versus high) and histopathological variables**

|  | **All cases** | | | **Adenocarcinoma** | | | **Squamous cell carcinoma** | | | **Other NSCLC** | | |
| --- | --- | --- | --- | --- | --- | --- | --- | --- | --- | --- | --- | --- |
|  | **n=437ᵃ** | | | **n=213** | | | **n=134ᵃ** | | | **n=90** | | |
|  | **OR** | **(95 % CI)** | **p** | **OR** | **(95 % CI)** | **p** | **OR** | **(95 % CI)** | **p** | **OR** | **(95 % CI)** | **p** |
| **Tumor diameter**  (≥35 mm vs <34 mm) | 0.93 | (0.64-1.36) | ns | 0.79 | (0.46-1.36) | ns | 2.03 | (0.97-4.25) | 0.059 | 0.61 | (0.26-1.45) | ns |
| **Histologic grade**  (high vs low) | 1.17 | (0.80-1.72) | ns | 1.64 | (0.94-2.83) | 0.079 | 0.78 | (0.40-1.55) | ns | 1.15 | (0.16-8.54) | ns |
| **BVI**  (present vs absent) | 1.26 | (0.81-1.96) | ns | 1.16 | (0.64-2.11) | ns | 1.42 | (0.59-3.44) | ns | 1.09 | (0.40-2.96) | ns |
| **LVI**  (present vs absent) | 0.65 | (0.41-1.03) | 0.065 | 0.73 | (0.39-1.34) | ns | 0.43 | (0.16-1.13) | 0.080 | 0.58 | (0.17-1.99) | ns |
| **Necrosis**  (present vs absent) | 0.98 | (0.63-1.51) | ns | 1.12 | (0.65-1.92) | ns | 1.88 | (0.45-7.84) | ns | 0.85 | (0.18-4.02) | ns |
| **Inflammation**  (severe vs mild/mod.) | 1.69 | (0.99-2.87) | 0.053 | 1.59 | (0.61-1.41) | ns | 1.79 | (0.79-4.02) | ns | 2.47 | (0.79-7.71) | ns |
| **Pleural invasion**  (present vs absent) | 1.43 | (0.93-2.21) | ns | 1.28 | (0.70-2.32) | ns | 1.42 | (0.59-3.44) | ns | 1.60 | (0.63-4.05) | ns |
| **Tumor stage**  (II-IV vs I) | 1.13 | (0.77-1.65) | ns | 0.87 | (0.50-1.51) | ns | 1.15 | (0.58-2.27) | ns | 1.94 | (0.82-4.60) | ns |

ᵃ1 case missing (staining)

n, number of patients; OR, odds ratio; CI, confidence interval; BVI, blood vessel invasion; LVI, lymph vessel invasion; p, p-values from Pearson’s chi-square test; ns, non-significant (p>0.10)

**Supplementary Table 2: Associations between Neuroserpin expression (present versus absent) and histopathological variables**

|  | **All cases** | | | **Adenocarcinoma** | | | **Squamous cell carcinoma** | | | **Other NSCLC** | | |
| --- | --- | --- | --- | --- | --- | --- | --- | --- | --- | --- | --- | --- |
|  | **n=438** | | | **n=213** | | | **n=135** | | | **n=90** | | |
|  | **OR** | **(95 % CI)** | **p** | **OR** | **(95 % CI)** | **p** | **OR** | **(95 % CI)** | **p** | **OR** | **(95 % CI)** | **p** |
| **Tumor diameter**  (≥35 mm vs <34 mm) | 1.64 | (1.06-2.54) | 0.026 | 1.30 | (0.73-2.30) | ns | 1.23 | (0.42-3.63) | ns | 1.88 | (0.68-5.23) | ns |
| **Histologic grade**  (high vs low) | 1.46 | (0.95-2.26) | 0.087 | 1.61 | (0.90-2.86) | ns | 1.26 | (0.44-3.62) | ns | 1.06 | (1.00-1.12) | ns |
| **BVI**  (present vs absent) | 1.19 | (0.71-2.00) | ns | 2.34 | (1.19-4.61) | 0.012 | 0.93 | (0.24-3.55) | ns | 0.38 | (0.13-1.17) | ns |
| **LVI**  (present vs absent) | 0.76 | (0.45-1.28) | ns | 0.88 | (0.47-1.68) | ns | 0.88 | (0.23-3.36) | ns | 0.77 | (0.19-3.20) | ns |
| **Necrosis**  (present vs absent) | 2.90 | (1.82-4.64) | <0.001 | 2.29 | (1.29-4.08) | 0.004 | 0.92 | (0.11-7.85) | ns | 1.55 | (0.28-8.71) | ns |
| **Inflammation**  (severe vs mild/mod.) | 1.49 | (0.78-2.85) | ns | 1.34 | (0.50-3.61) | ns | 0.62 | (0.19-1.93) | ns | 2.28 | (0.47-10.97) | ns |
| **Pleural invasion**  (present vs absent) | 2.05 | (1.17-3.59) | 0.011 | 2.65 | (1.33-5.28) | 0.005 | 3.59 | (0.45-28.62) | ns | 1.68 | (0.50-5.66) | ns |
| **Tumor stage**  (II-IV vs I) | 1.04 | (0.67-1.62) | ns | 1.13 | (0.64-2.02) | ns | 0.91 | (0.32-2.59) | ns | 1.04 | (0.37-2.91) | ns |

n, number of patients; OR, odds ratio; CI, confidence interval; BVI, blood vessel invasion; LVI, lymph vessel invasion; p, p-values from Pearson’s chi-square test; ns, non-significant (p>0.10)

**Supplementary Table 3: Associations between L1CAM expression (present versus absent) and histopathological variables**

|  | **All cases** | | | **Adenocarcinoma** | | | **Squamous cell carcinoma** | | | **Other NSCLC** | | |
| --- | --- | --- | --- | --- | --- | --- | --- | --- | --- | --- | --- | --- |
|  | **n=438** | | | **n=213** | | | **n=135** | | | **n=90** | | |
|  | **OR** | **(95 % CI)** | **p** | **OR** | **(95 % CI)** | **p** | **OR** | **(95 % CI)** | **p** | **OR** | **(95 % CI)** | **p** |
| **Tumor diameter**  (≥35 mm vs <34 mm) | 1.42 | (0.97-2.08) | 0.068 | 1.42 | (0.83-2.45) | ns | 1.26 | (0.61-2.60) | ns | 1.05 | (0.44-2.51) | ns |
| **Histologic grade**  (high vs low) | 2.02 | (1.38-2.97) | <0.001 | 2.13 | (1.23-3.69) | 0.007 | 2.08 | (1.03-4.20) | 0.039 | 1.08 | (1.00-1.16) | ns |
| **BVI**  (present vs absent) | 2.06 | (1.31-3.24) | 0.002 | 3.56 | (1.92-6.59) | <0.001 | 1.85 | (0.71-4.82) | ns | 0.89 | (0.32-2.45) | ns |
| **LVI**  (present vs absent) | 1.65 | (1.02-2.65) | 0.038 | 1.76 | (0.95-3.27) | 0.071 | 1.71 | (0.65-4.49) | ns | 3.48 | (0.71-16.99) | ns |
| **Necrosis**  (present vs absent) | 2.96 | (1.88-4.67) | <0.001 | 3.81 | (2.12-6.38) | <0.001 | 1.16 | (0.30-4.53) | ns | 0.25 | (0.03-2.19) | ns |
| **Inflammation**  (severe vs mild/mod.) | 1.13 | (0.67-1.89) | ns | 0.94 | (0.38-2.34) | ns | 0.94 | (0.42-2.18) | ns | 1.14 | (0.38-3.43) | ns |
| **Pleural invasion**  (present vs absent) | 1.41 | (0.91-2.17) | ns | 2.67 | (1.46-4.87) | 0.001 | 0.96 | (0.39-2.34) | ns | 0.61 | (0.24-1.55) | ns |
| **Tumor stage**  (II-IV vs I) | 1.39 | (0.95-2.04) | 0.090 | 1.94 | (1.01-3.40) | 0.021 | 0.86 | (0.43-1.71) | ns | 1.47 | (0.61-3.51) | ns |

n, number of patients; OR, odds ratio; CI, confidence interval; BVI, blood vessel invasion; LVI, lymph vessel invasion; p, p-values from Pearson’s chi-square test; ns, non-significant (p>0.10)

**Supplementary Table 4: Associations between SerpinB2 expression (low versus high) with different sites of metastasis**

|  | **All cases** | | | **Adenocarcinoma** | | | **Squamous cell carcinoma** | | | **Other NSCLC** | | |
| --- | --- | --- | --- | --- | --- | --- | --- | --- | --- | --- | --- | --- |
|  | **N=437ᵇ** | | | **N=213** | | | **N=134ᵇ** | | | **N=90** | | |
|  | **OR** | **(95 % CI)** | **p** | **OR** | **(95 % CI)** | **p** | **OR** | **(95 % CI)** | **p** | **OR** | **(95 % CI)** | **p** |
| **Liverᶜ** | 1.52 | (0.78-2.97) | ns | 1.73 | (0.63-4.73) | ns | 1.16 | (0.28-4.83) | ns | 1.40 | (0.42-4.68) | ns |
| **Adrenals** | 1.15 | (0.49-2.68) | ns | 0.74 | (0.27-2.04) | ns | NA | | | 2.27 | (0.42-12.36) | ns |
| **Brain** | 1.42 | (0.85-2.37) | ns | 0.83 | (0.43-1.59) | ns | 3.70 | (0.72-19.03) | ns | 2.67 | (0.86-8.29) | ns |
| **Bone** | 1.09 | (0.63-1.86) | ns | 1.08 | (0.52-2.23) | ns | 1.96 | (0.61-6.33) | ns | 0.48 | (0.14-1.60) | ns |
| **Skin** | 1.03 | (0.34-3.12) | ns | 1.94 | (0.37-10.23) | ns | 0.37 | (0.04-3.67) | ns | 0.85 | (0.05-14.05) | ns |
| **Other metastasesᵃ** | 0.75 | (0.41-1.38) | ns | 0.52 | (0.23-1.17) | ns | 0.91 | (0.23-3.55) | ns | 1.32 | (0.35-5.05) | ns |

ᵃOther metastases, intrathoracic, distant lymph nodes, kidneys, spleen, gastrointestinal tract; ᵇ1 case missing (staining); ᶜFor number of cases in each subgroup, see Table 3

n, number of patients; OR, odds ratio; CI, confidence interval; ns, not significant (p>0.10); NA, not applicable (no adrenal metastases in this subgroup); p-values from Pearson’s chi-square test

**Supplementary Table 5: Associations between Neuroserpin expression (present versus absent) with different sites of metastasis**

|  | **All cases** | | | **Adenocarcinoma** | | | **Squamous cell carcinoma** | | | **Other NSCLC** | | |
| --- | --- | --- | --- | --- | --- | --- | --- | --- | --- | --- | --- | --- |
|  | **N=438** | | | **N=213** | | | **N=135** | | | **N=90** | | |
|  | **OR** | **(95 % CI)** | **p** | **OR** | **(95 % CI)** | **p** | **OR** | **(95 % CI)** | **p** | **OR** | **(95 % CI)** | **p** |
| **Liverᵇ** | 0.89 | (0.43-1.84) | ns | 0.55 | (0.21-1.41) | ns | 1.08 | (1.03-1.14) | ns | 1.61 | (0.33-7.99) | ns |
| **Adrenals** | 0.49 | (0.20-1.16) | 0.096 | 0.49 | (0.17-1.35) | ns | NA | | | 1.69 | (0.19-14.94) | ns |
| **Brain** | 0.60 | (0.33-0.98) | 0.040 | 0.71 | (0.37-1.38) | ns | 0.95 | (0.11-8.23) | ns | 0.64 | (1.20-2.09) | ns |
| **Bone** | 1.15 | (0.61-2.18) | ns | 1.44 | (0.65-3.17) | ns | 1.70 | (0.21-14.01) | ns | 0.89 | (0.22-3.62) | ns |
| **Skin** | 0.52 | (0.17-1.61) | ns | 0.69 | (0.15-3.15) | ns | 0.39 | (0.38-4.01) | ns | 0.26 | (0.02-4.38) | ns |
| **Other metastasesᵃ** | 0.96 | (0.48-1.92) | ns | 1.35 | (0.57-3.24) | ns | 1.09 | (0.13-9.34) | ns | 0.59 | (0.14-2.55) | ns |

ᵃOther metastases, intrathoracic, distant lymph nodes, kidneys, spleen, gastrointestinal tract; ᵇFor number of cases in each subgroup, see Table 3

n, number of patients; OR, odds ratio; CI, confidence interval; ns, not significant (p>0.10); NA, not applicable (no adrenal metastases in this subgroup); p-values from Pearson’s chi-square test

**Supplementary Table 6: Associations between L1CAM expression (present versus absent) with different sites of metastasis**

|  | **All cases** | | | **Adenocarcinoma** | | | **Squamous cell carcinoma** | | | **Other NSCLC** | | |
| --- | --- | --- | --- | --- | --- | --- | --- | --- | --- | --- | --- | --- |
|  | **N=438** | | | **N=213** | | | **N=135** | | | **N=90** | | |
|  | **OR** | **(95 % CI)** | **p** | **OR** | **(95 % CI)** | **p** | **OR** | **(95 % CI)** | **p** | **OR** | **(95 % CI)** | **p** |
| **Liverᵇ** | 1.33 | (0.69-2.55) | ns | 3.99 | (1.38-11.52) | 0.007 | 0.53 | (0.14-2.08) | ns | 0.48 | (0.15-1.58) | ns |
| **Adrenals** | 1.00 | (0.43-2.32) | ns | 0.75 | (0.26-2.15) | ns | NA | | | 4.04 | (0.47-35.12) | ns |
| **Brain** | 0.99 | (0.60-1.64) | ns | 1.15 | (0.60-2.20) | ns | 1.17 | (0.27-5.12) | ns | 0.96 | (0.33-2.79) | ns |
| **Bone** | 1.22 | (0.71-2.09) | ns | 1.52 | (0.74-3.12) | ns | 1.13 | (0.35-3.65) | ns | 0.99 | (0.30-3.31) | ns |
| **Skin** | 2.10 | (0.64-6.91) | ns | 3.29 | (0.63-17.33) | ns | 0.69 | (0.09-5.04) | ns | 1.04 | (0.99-1.09) | ns |
| **Other metastasesᵃ** | 1.26 | (0.69-2.33) | ns | 1.55 | (0.70-3.44) | ns | 0.86 | (0.22-3.36) | ns | 1.51 | (0.36-6.27) | ns |

ᵃOther metastases, intrathoracic, distant lymph nodes, kidneys, spleen, gastrointestinal tract; ᵇFor number of cases in each subgroup, see Table 3

n, number of patients; OR, odds ratio; CI, confidence interval; ns, not significant (p>0.10); NA, not applicable (no adrenal metastases in this subgroup); p-values from Pearson’s chi-square test

**Supplementary Table 7: Demographic and clinico-pathologic data for the matched primary-metastasis series of non-small cell lung carcinoma (n=43)**

|  | **n** | **(%)** |
| --- | --- | --- |
| **Age (median)** |  |  |
| <=68 years | 27 | (62.8) |
| >69 years | 16 | (37.2) |
| **Sex** |  |  |
| Male | 27 | (62.8) |
| Female | 16 | (37.2) |
| **Histologic type** |  |  |
| Adenocarcinoma | 22 | (51.2) |
| Squamous cell carcinoma | 9 | (20.9) |
| Other NSCLC | 12 | (27.9) |
| **Histologic grade** |  |  |
| Well differentiated | 3 | (7.0) |
| Mod. differentiated | 12 | (27.9) |
| Poorly differentiated | 19 | (44.2) |
| Undifferentiated | 9 | (20.9) |
| **Site of metastatic biopsy** |  |  |
| Liver | 2 | (4.7) |
| Brain | 11 | (25.6) |
| Bone | 6 | (14.0) |
| Skin | 7 | (16.3) |
| Other | 17 | (39.5) |
| **Status at latest observation** |  |  |
| Recovered | 4 | (9.3) |
| Alive with disease recurrence | 1 | (2.3) |
| Dead because of lung cancer | 33 | (76.7) |
| Dead of other causes | 5 | (11.7) |

n, number of cases

**Supplementary Table 8A: Frequency distribution for expression of SerpinB2, Neuroserpin and L1CAM in matched metastatic biopsies by site (n=43)**

|  |  | **Liver** | | **Brain** | | **Bone** | | **Skin** | | **Other sitesᵃ** | |  |
| --- | --- | --- | --- | --- | --- | --- | --- | --- | --- | --- | --- | --- |
|  |  | **n** | **(%)** | **n** | **(%)** | **n** | **(%)** | **n** | **(%)** | **n** | **(%)** | **p** |
| **SerpinB2** | Low | 0 | (0) | 7 | (43.8) | 2 | (12.5) | 4 | (25.0) | 3 | (18.8) |  |
|  | High | 2 | (7.4) | 4 | (14.8) | 4 | (14.8) | 3 | (11.1) | 14 | (51.9) | 0.068 |
| **Neuroserpin** | Absent | 0 | (0) | 3 | (33.3) | 0 | (0) | 1 | (11.1) | 5 | (55.6) |  |
|  | Present | 2 | (5.9) | 8 | (23.5) | 6 | (17.6) | 6 | (17.6) | 12 | (35.3) | 0.522 |
| **L1CAMᵇ** | Absent | 1 | (5.3) | 7 | (36.8) | 2 | (10.5) | 3 | (15.8) | 6 | (31.6) |  |
|  | Present | 1 | (4.3) | 4 | (17.4) | 4 | (17.4) | 4 | (17.4) | 10 | (43.5) | 0.761 |

ᵃOther sites= intrathoracic, distant lymph nodes, kidneys, spleen, gastrointestinal; ᵇ1 missing case (no tumor left), p-values from Pearson’s chi-square test

N= number of cases

**Supplementary Table 8B: Frequency distribution for expression of SerpinB2, Neuroserpin and L1CAM in matched brain metastases versus expression in other metastatic sites combined (n=43)**

|  |  | **Brain** | | **Other sites combinedᵃ** | |  |
| --- | --- | --- | --- | --- | --- | --- |
|  |  | **n** | **(%)** | **n** | **(%)** | **p** |
| **SerpinB2** | Low | 7 | (43.8) | 9 | (56.3) |  |
|  | High | 4 | (14.8) | 23 | (85.2) | 0.068 |
| **Neuroserpin** | Absent | 3 | (33.3) | 6 | (66.7) |  |
|  | Present | 8 | (23.5) | 26 | (76.5) | 0.672 |
| **L1CAMᵇ** | Absent | 7 | (36.8) | 12 | (63.2) |  |
|  | Present | 4 | (17.4) | 19 | (73.8) | 0.180 |

ᵃOther sites= intrathoracic, distant lymph nodes, kidneys, spleen, gastrointestinal; ᵇ1 missing case (no tumor left), p-values from Pearson’s chi-square test

N= number of cases

**Supplementary Table 9: Univariate survival analysis for SerpinB2, Neuroserpin and L1CAM (Log rank-test for comparison between groups) in all cases and in each histological subgroup**

|  | **All cases** | | **Adenocarcinoma** | | **Squamous cell carcinoma** | | **Other NSCLC** | |
| --- | --- | --- | --- | --- | --- | --- | --- | --- |
|  | **N=438** | | **N=213** | | **N=135** | | **N=90** | |
|  | **LCSS** | **DFS** | **LCSS** | **DFS** | **LCSS** | **DFS** | **LCSS** | **DFS** |
| **SerpinB2¹** | 0.280 | 0.653 | 0.017 | 0.023 | 0.304 | 0.444 | 0.739 | 0.365 |
| **Neuroserpin** | 0.930 | 0.728 | 0.872 | 0.898 | 0.137 | 0.173 | 0.688 | 0.385 |
| **L1CAM** | 0.312 | 0.586 | 0.435 | 0.197 | 0.562 | 0.509 | 0.399 | 0.551 |

Numbers in cells are p-values from these analyses

¹ 1 missing case (staining evaluation not possible)

n, number of patients; LCSS, lung cancer specific survival; DFS, disease-free survival

**Supplementary Table 10A: Univariate and multivariate analysis (Cox proportional hazards method) for stage I adenocarcinomas (n=90) with regards to lung cancer specific survival and SerpinB2 expression**

| **Variable** | **Categories** | **Univariate** | | | **Multivariate** | | |
| --- | --- | --- | --- | --- | --- | --- | --- |
|  |  | **HR** | **(95% CI)** | **p** | **HR** | **(95% CI)** | **p** |
| **Age** | ≤68 years | 1.0 |  |  | NI | | |
|  | >68 years | 1.8 | (0.73-4.32) | 0.203 |  |  |  |
| **Sex** | Female | 1.0 |  |  | 1.0 |  |  |
|  | Male | 5.3 | (1.91-14.53) | 0.001 | 5.1 | (1.77-14.70) | 0.003 |
| **SerpinB2** | High | 1.0 |  |  | 1.0 |  |  |
|  | Low | 2.6 | (1.06-6.18) | 0.038 | 2.6 | (1.03-6.58) | 0.043 |
| **Histologic grade** | Low | 1.0 |  |  | NI | | |
|  | High | 1.4 | (0.59-3.31) | 0.453 |  |  |  |
| **Blood vessel invasion** | Absent | 1.0 |  |  | NI | | |
|  | Present | 2.3 | (0.84-6.55) | 0.105 |  |  |  |
| **Lymph vessel invasion** | Absent | 1.0 |  |  | NI | | |
|  | Present | 0.7 | (0.09-5.39) | 0.726 |  |  |  |
| **Necrosis** | Absent | 1.0 |  |  | 1.0 |  |  |
|  | Present | 2.5 | (1.03-6.33) | 0.044 | 1.5 | (0.54-3.86) | 0.460 |
| **Inflammation** | Mild/moderate | 1.0 |  |  | NI | | |
|  | Severe | 1.1 | (0.24-4.59) | 0.953 |  |  |  |

HR, hazard ratio; CI, confidence interval; NI, not included

**Supplementary Table 10B: Univariate and multivariate analysis (Cox proportional hazards method) for stage I squamous cell carcinomas (n=62) with regards to lung cancer specific survival and SerpinB2 expression**

| **Variable** | **Categories** | **Univariate** | | | **Multivariate** | | |
| --- | --- | --- | --- | --- | --- | --- | --- |
|  |  | **HR** | **(95% CI)** | **p** | **HR** | **(95% CI)** | **p** |
| **Age** | ≤68 years | 1.0 |  |  | NI | | |
|  | >68 years | 0.6 | (0.16-1.88) | 0.344 |  |  |  |
| **Sex** | Female | 1.0 |  |  | NI | | |
|  | Male | 3.0 | (0.39-22.99) | 0.293 |  |  |  |
| **SerpinB2** | Low | 1.0 |  |  | 1.0 |  |  |
|  | High | 3.7 | (1.12-12.29) | 0.032 | 3.5 | (1.03-11.74) | 0.044 |
| **Histologic grade** | Low | 1.0 |  |  | NI | | |
|  | High | 1.4 | (0.46-4.13) | 0.560 |  |  |  |
| **Blood vessel invasion** | Absent | 1.0 |  |  | 1.0 |  |  |
|  | Present | 4.2 | (1.10-16.34) | 0.036 | 3.7 | (0.92-14.55) | 0.066 |
| **Lymph vessel invasion** | Absent | 1.0 |  |  | NI | | |
|  | Present | 1.5 | (0.19-11.56) | 0.705 |  |  |  |
| **Necrosis** | Absent | 1.0 |  |  | NI | | |
|  | Present | 1.5 | (0.19-11.35) | 0.718 |  |  |  |
| **Inflammation** | Mild/moderate | 1.0 |  |  | NI | | |
|  | Severe | 0.8 | (0.23-3.08) | 0.799 |  |  |  |

HR, hazard ratio; CI, confidence interval; NI, not included
